# What are the palliative and end-of-life care needs and preferences of people with cancer from culturally and linguistically diverse (CALD) backgrounds?: A systematic review

**DOI:** 10.1007/s00520-026-11341-2

**Published:** 2026-10-29

**Authors:** Sadhvi S. S. Naresh, Ashfaq Chauhan, Bronwyn Newman, Reema Harrison

**Affiliations:** 1 Faculty of Medicine, Health & Human Sciences, Macquarie University, North Ryde, NSW Australia; 2 Australian Institute of Health Innovation, Macquarie University, North Ryde, New South Wales Australia

**Keywords:** Ethnic minority, Culturally and linguistically diverse populations, Cancer, Palliative care, End of life care, Systematic review

## Abstract

**Purpose:**

Goal-concordant care is critical to supporting patients at the end of life (EOL), and palliative care is a key facet of this. The study aims to (1) synthesize the evidence available on the EOL and palliative care (PC) needs and preferences of people with cancer from culturally and linguistically diverse (CALD) backgrounds and (2) explore what elements of service provision are facilitators or barriers to service engagement and goal-concordant care.

**Methods:**

A systematic review was conducted. The search strategy was applied to five electronic databases to identify relevant articles published between January 2003 and September 2025. Quality assessment was performed using the Quality Assessment for Diverse Studies (QuADS) tool. A narrative synthesis was led by one reviewer with the support of a wider team.

**Results:**

A total of 6017 titles were identified. After duplicate removal and screening, 20 studies were included in the review. Fourteen studies reported EOL care and six reported PC encounters. People from CALD backgrounds identified the following needs: (1) maintaining positivity, (2) maintaining decision-making autonomy, (3) involving family/caregivers in decision-making, and (4) trust in the provider–patient relationship. Factors which influenced patient engagement and care preferences were (1) family involvement, (2) faith/spirituality, and (3) understanding of services.

**Conclusion:**

This review illustrates the distinct care complexities for patients from CALD backgrounds. These findings underscore the importance of avoiding cultural generalizations and involving consumers in system planning. System-level changes, including community consultation, and improved access to culturally/linguistically appropriate resources are needed to support service engagement.

**Supplementary Information:**

The online version contains supplementary material available at https://doi.org/10.1007/s00520-026-11341-2.

## Background

People with advanced cancer often have a significant symptom burden and a need for physical and psychosocial support, especially as they approach end of life (EOL) [1]. Palliative care (PC) is an important strategy to support delivery of optimal care to improve patients’ quality of life, both at the EOL and at other times in a patient’s care management journey, by providing goal-concordant care [2, 3]. Early referral to PC services is internationally recommended as it improves patient outcomes and reduces hospital costs and utilization [4, 5]. However, in practice, PC referrals are not routine, and EOL services are underutilized [6].

The term culturally and linguistically diverse (CALD) is used in Australia to describe people with non-Western “languages, ethnic backgrounds, traditions, societal structures, and religions” as well as those born in countries other than “main English speaking” countries [7]. Needs and preferences for both EOL and PC are often influenced by the social, cultural, and religious values of patients [8]. People from immigrant backgrounds in Australia often utilize healthcare services less often, face more barriers to access, and experience more unmet healthcare needs [9]. As noted in Khartri et al.’s 2022 scoping review, this may be due to additional barriers at individual, community, and system levels, for example, acculturation or poor health system literacy [10]. Australia is a multicultural society, with nearly 30% of the population born overseas [11]. There is considerable inter- and intraethnic diversity among CALD populations. It is important to understand what factors may influence needs and preferences for care among these populations when creating individualized plans which maintain patient autonomy, dignity, and emotional wellbeing [12].

As noted by Bazargan-Hejazi’s scoping review of PC and hospice disparities, there was both dissatisfaction and a disproportionate underutilization of PC by people with cancer from CALD backgrounds, with these patients more likely to undergo unnecessary interventions [13]. Furthermore, as noted by Mack et al., care received was often not in line with patients’ expressed goals of care [14]. This mismatch between patient preferences and experiences highlights a troubling disconnect between healthcare consumers, institutions, and providers which could undermine patient trust in services [15]. Understanding what facilitates and hinders goal-aligned service provisions is necessary to enable care that is patient-centered and guide culturally congruent healthcare. Developing individualized care plans which address the requirements of people from CALD backgrounds is necessary in helping to maintain patient autonomy, dignity, and emotional wellbeing [12]. This is especially true towards the EOL when people may feel a loss of agency [16] or experience an increase in physical, social, or psychological needs [17].

However, there is a paucity of synthesized evidence identifying the dimensions of the palliative and EOL care needs and preferences of people with cancer from CALD backgrounds. Prior research has often focused on (1) populations with specific sociodemographic characteristics which influence care provision [18], (2) provider perspectives on pitfalls of care [19], (3) preferences regarding facets of care [20] or ways to express/document these preferences [21], or (4) country-specific perspectives which may not be generalizable [22, 23]. A similar systematic review exploring minority patients’ EOL care (EOLC) preferences was conducted by Lopresti et al. in 2014. This study found African American patients had inadequate knowledge of hospice services, and the EOLC provided was inconsistent with preferences. However this study was conducted over 10 years ago, did not investigate PC, and excluded non-American patients [24].

This systematic review will aim to (1) identify the needs and preferences of people with cancer from CALD backgrounds accessing end of life/palliative care and (2) explore what elements of service provision function as facilitators or barriers to service engagement and value aligned care. The results of this review will be pivotal for healthcare providers and administrators to design and deliver person-centered and culturally appropriate end of life cancer care.

## Methods

### Design

A systematic review was conducted and reported using the 2020 Preferred Reporting Items for Systematic Reviews and Meta-Analyses (PRISMA) guidelines [25] (Appendix 1). The study protocol was prospectively registered on PROSPERO (CRD42024500987).

### Search strategy and data sources

Five databases (Medline, PsycInfo, CINAHL, Embase, Scopus) were searched. A medical librarian and research team familiar with this field (AC, BN) assisted in search strategy development. The search was updated in September 2025 to account for new articles published after the initial search date. The search strategy, shown in Appendix 2, was applied using Medical Subject Headings (MeSH) and keywords for five concepts: (1) cultural/linguistic diversity, (2) palliative end of life care, (3) cancer, (4) voluntary assisted dying (VAD), and (5) patient preferences, needs, and experiences. For the purposes of this study, patient needs were defined as “the physiological, safety, belonging, self-esteem, and self-actualization requirements for wellbeing” [26]. Preferences were defined as “patient’s preferred choices based on their attitudes and perceptions” [27]. Experiences were defined as “the range of subjectively reported interactions a patient has with the healthcare system, including but not limited to access to care and information, communication with healthcare providers, and support services” [28].

### Inclusion and exclusion criteria

We included peer-reviewed primary studies published in English from 01 January 2003 to 07 September 2025. The date range included studies published in the 20 years prior to study design, in line with a previous systematic review in this field [24]. Publications were excluded if they were not from countries within the Organisation for Economic Co-operation and Development or if they focused on pediatric populations. Studies focusing on people from Australian Indigenous backgrounds were excluded due to their unique position and experiences as first nations people [29]. Studies that reported only caregiver/physician needs or preferences were excluded. Studies focusing on only advance care planning (ACP) were excluded as this systematic review examined patient needs and preferences, not how they document them. Given the evolving inclusion of VAD as part of the EOLC sphere, studies reporting on these experiences were included. Appendix 3 gives the inclusion and exclusion criteria.

### Screening and quality appraisal

After removing duplicates, titles and abstracts of retrieved articles were independently screened by two authors (SSSN, SA), using selection criteria given in Appendix 3. Full-text review was conducted independently by all authors (SSSN, BN, AC) and conflicts resolved by a third author (AC, BN). Reference lists of included articles were checked for additional eligible studies.

Given the heterogeneity of study types and methods, the Quality Assessment for Diverse Studies (QuADS) checklist [30] was used for quality appraisal. The QuADS tool comprises 13 domains, with three authors (SSSN, AC, BN) independently providing a score for each domain from zero to three. The QuADS checklist can be found in Appendix 4. The kappa test [31] was used to determine interrater reliability between the three reviewers.

### Data extraction and synthesis

Following full-text review, data was extracted by one author (SSSN). The following data were extracted using a data extraction form: study characteristics, including author, date, country of study, study center, and number of participants; participant characteristics, including age, gender, ethnic background, cancer type, and cancer stage; study methods and key themes. Given the heterogeneity of study methods and outcomes, results were categorized and analyzed through an inductive narrative synthesis which identified recurring themes through tabulation and grouping of similar findings in an inductive manner and a textual approach used to summarize the findings [31]. Three authors (SSSN, AC, BN) independently participated in full-text review of 153 articles. Results were initially extracted by one author (SSSN) and later reviewed by the other authors (AC, BN) to ensure accuracy.

## Results

**Fig. 1:**
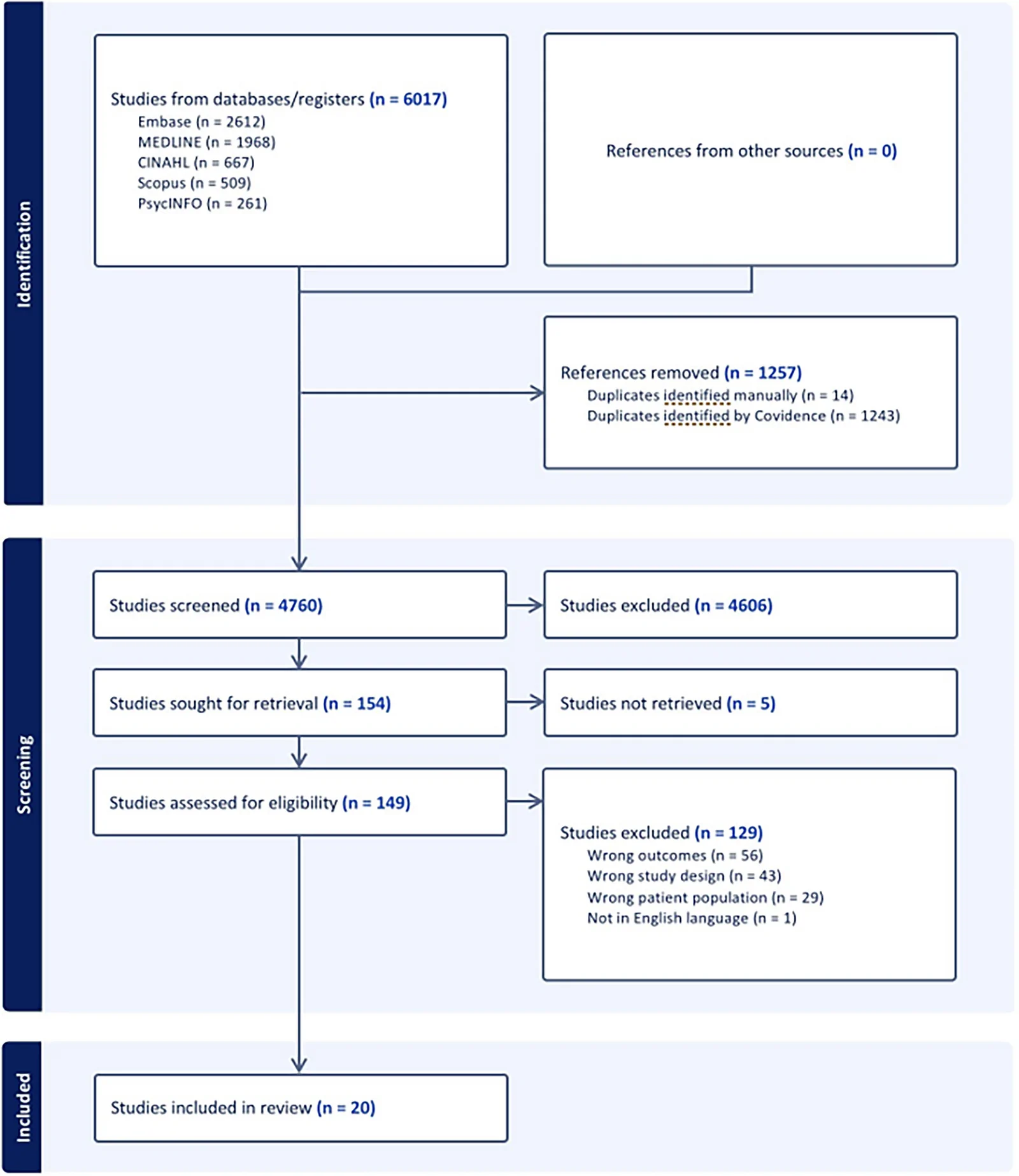
PRISMA flowchart of included studies

After 1257 duplicates were removed, 4760 articles underwent title and abstract screening. Following full-text review of 153 articles, 20 articles were included, as shown in Fig. 1.

### Study characteristics

Twelve qualitative [32, 34, 36, 38, 40–42, 44, 45, 48–50], six quantitative [33, 35, 37, 39, 43, 46], and two mixed methods [48, 51] studies that were published between 2003 and 2022 were included. Studies originated from the USA (15) [32, 33, 35–39, 41–44, 46–48, 51], Canada (2) [45, 49], UK (1) [50], Australia (1) [40], and New Zealand (1) [34]. Study data were collected using interviews (17), surveys (2), and focus groups (1). Out of 20 studies included, 14 [32, 37, 41, 43, 44, 46–48, 50, 51] explored EOLC interactions across hospital, outpatient, community, and hospice settings. Six studies [38–40, 42, 45, 49] investigated PC encounters. Half of the 20 included studies [32, 33, 38, 39, 43, 44, 46–48, 51] aimed to investigate patients’ care preferences; the other half explored patients’ experiences [34–37, 40–42, 45, 49, 50]. Baseline study characteristics and summary of study findings are presented in Table 1.

**Table 1:** Summary of study findings

Author	Country and setting	Aim(s)	Sample	Study design and methods	Relevant findings
Campbell, 2010	USA, 2 hospice programs, 2 × cancer centers	What individual, clinical, and social factors influence African Americans who have been diagnosed with a life-limiting illness to choose hospice or other options for EOL care?	*N* = 7 patients African American patients with stage III/IV cancer Aged 53–87 with KPS scores ≥ 70	Qualitative Data collection: guided interviews Data analysis: thematic analysis	3 main types of EOL decisions: decisions about treatment, the source of care, and communication Most participants had family members or caregivers who were involved in the decision-making Involvement of family or caregivers in decision-making varied, depending on availability, geographic proximity, and the health condition of the caregivers A major source of conflict was the physician did not ask the patient to bring family to the office visit when hospice was discussed … [patient’s] anger that [the doctor] failed to include her family Patients did not think they were sick enough initially to begin hospice Participants followed the recommendations of the physician but not without personal reflection about the impact of the decisions on their lives African Americans with advanced cancer had their own metric for determining the impact of treatment on their lives and making EOL decisions such as enrolling in hospice but they did not blindly follow the physician’s order
Fishman, 2010	USA, 6 × oncology clinics	To define and compare preferences for cancer treatment and perceived needs for hospice services among African American patients and White patients	*N* = 283 patients 81 African American, 202 White patients Breast (*N* = 78), gastrointestinal (*N* = 50), hematological (*N* = 35), lung (*N* = 35), prostate (*N* = 34), oropharynx (*N* = 27), genitourinary (*N* = 17), sarcoma (*N* = 5), melanoma (*N* = 2)	Quantitative Data collection: conjoint interviews Data analysis: bivariate analysis, logistic regression Wilcoxon rank-sum test, linear regression	African American patients had stronger preferences for aggressive cancer treatment compared with White patients African American patients also were less likely to refuse all cancer treatment even if it were associated with an “almost 100%” probability of surviving for 6 months Compared with White patients, African American patients reported greater needs for all hospice services
Frey, 2013	New Zealand, hospice patients	To identify challenges to the use of hospice services for Maori, Pacific, and Asian patients within the context of one district health board To explore the attitudes of Maori, Pacific, and Asian patients and their family/whanau towards hospice services care To identify the reasons that eligible patients of Maori, Pacific, or Asian origin did not utilize the hospice services under study	*N* = 37 patients Aged > 18 Included New Zealand European, Maori, Samoan, Cook Island Maori, Tongan, Niuean, Fijian, Fijian Indian, Tokelaun, Korean, Chinese, and Malaysian participants	Qualitative Data collection: semistructured interviews Data analysis: thematic analysis	Patients and families often reported a lack of familiarity with the role of hospice services or the services available (“I’ve never heard about palliative care… Hospice information was given by specialist without any explanation.) Patients do not understand the flexibility that is allowed in hospice services care with regard to following cultural and spiritual practices Patients found hospice terminology difficult to understand Barriers to information access were responsible in part for the lack of understanding concerning both the meaning as well as the range of services available. These barriers were created through issues with language as well as by the cultural presumptions held by some healthcare providers Language challenges identified by participants included both translation (rendering written material accurately into a target language) and interpretation (transposing spoken messages from one language to another) Tongan patients did not feel comfortable with an interpreter. They want[ed]t to talk straight to the [doctor] Patients felt some physicians made cultural assumptions, limited access to hospice service information on the “attitude that [patients] won’t use it anyway” Misinformation or misunderstandings about the nature of palliative care and hospice service (e.g., nature of hospice services, the quality of services available, inclusiveness of the services) were reported A mistrust of the health system and the potential financial impact of use also existed Hospice was perceived as a place rather than a philosophy of care There was a perception that hospice services are only available for persons with an inability to pay
Garrido, 2014	USA, outpatient clinics from 5 states	To understand the degree to which EOL values and preferences are associated with rates of do-not-resuscitate (DNR) orders within non-Latino White, Black, and Latino patients with advanced cancer	*N* = 606 patients Advanced cancer and distant metastases or cancer refractory to chemotherapy within 4 months of death *N* = 437 non-Hispanic White patients, *N* = 93 Black patients, *N* = 76 Latino patients	Quantitative Data collection: interviews Data analysis: bivariate analysis	45% of White patients compared to 25% of Latino and Black patients reported having a DNR White patients with DNR orders were significantly less likely to prefer life extension over pain relief Fewer individuals with DNR orders had high levels of positive religious coping Among Latino patients, terminal illness acknowledgment was associated with a higher likelihood of having a DNR order The correlation between preferences against life-prolonging care and the increased likelihood of advance care planning is consistent across racial/ethnic groups
Glaser, 2020	USA, safety net hospital and free cancer care clinic	To explore how low-income Latina immigrants with breast cancer relate to their support networks and cope with advanced disease	*N* = 13 patients Aged 40–67 All patients born in Latin America, mean number of years in America was 24	Qualitative Data collection: translator assisted semistructured interviews Data analysis: thematic analysis	All patients described staying positive as a method of coping with illness Many patients thought God was the “ultimate decider of their fate,” but they also viewed doctors as “instruments of God” and believed staying positive and adhering to medical treatments as “part of their role” The importance of positivity was related to the belief that negative thinking worsens health Patients also stayed positive to protect loved ones. Some patients expressed appreciation when members of their support networks maintained positivity, stating that this helped keep them motivated Many patients reported that they preferred not to discuss death because it made them feel sad or anxious. Others chose not to in order to spare loved ones from distress. Some patients felt a desire to discuss these topics but met resistance Multiple patients expressed desire and gratitude for the opportunity to talk about EOL issues in a supportive context. Study participants felt more trust for providers whom they perceived as having adequate time and a warm demeanor Patients generally preferred bilingual providers, describing an increased sense of comfort and trust. Some patients described “getting by” with limited English or ad hoc interpreters, e.g., administrative staff or children
Halpern, 2017	USA, SEER-CAHPS survey respondents	To describe patient experiences with physicians, health plans and care and medical care and assess associations between patient characteristics and these experiences among individuals with cancer who completed the survey within 1 year before their death	*N* = 160,959 patients Aged > 66 Died within 12 months of survey Breast (*N* = 554), colorectal (*N* = 560), lung (*N* = 773), prostate (*N* = 756), other (*N* = 2046) Interviewed: White, Black, Asian, Hispanic, and “other” patients	Quantitative Data collection: survey Data analysis: multivariate logistic regression	Asian and Hispanic patients were found to be significantly less likely to indicate excellent experience for getting needed care
Jia, 2022	USA, cancer center	Exploring the preferences of Chinese patients with advanced cancer and their caregivers for ACP	*N* = 20 patients Patients with gastrointestinal (*N* = 8), lung (*N* = 7), and breast (*N* = 5) cancer Mean age 57.5 *N* = 18 born in China, *N* = 2 born in the USA; median US residency of 17.5 years	Qualitative Data collection: exploratory qualitative semistructured interviews Data analysis: thematic analysis	Patients trust clinicians to guide ACP conversations and preferred clinicians to lead ACP conversations Patients thought physicians would gauge the appropriateness to offer ACP based on their health status Patients often selectively choose their confidants based on perceived psychological burden Some patients preferred clinicians to indirectly discuss and infer care goals and priorities: “They [physicians] should be able to interpret these things and catch it without speaking directly about it” Patients appreciate focusing on actionable short-term goals, e.g., “maintain a positive mood” Palliative care was an unfamiliar and misperceived service Importance of self-advocacy and being explicit about communication preferences, and this explicitness being at-times contrary to cultural values was identified by patients
Jonnalagadda, 2012	USA, 4 × medical centers	Differences in beliefs and attitudes re lung cancer treatment	*N* = 335 patients Lung cancer patients aged > 18 *N* = 70 Black patients, *N* = 67 Hispanic patients, *N* = 198 nonminority patients	Quantitative Data collection: questionnaire Data analysis: self-regulation model; chi-squared/Fisher exact test	Black and Hispanic patients were more likely than nonminority patients to have fatalistic beliefs, i.e., bad things are meant to be and should be accepted and that “everything is past of God’s plan Significant misperceptions among minorities regarding ACP identified - Hispanics more likely to feel it is preferable to have family make medical decisions - Blacks more likely to feel living wills exclude receiving cancer treatment Blacks were more likely to hold faith-driver beliefs that life sustaining treatment should be continued as “only God can decide when it is time for death” Minority patients were more likely to believe hospice was restricted to patients expected to die in a few days
Kirby, 2022	Australia, hospital oncology/PC patients	To explore attitudes towards, and understandings and experiences of, end of life care among CALD patients and their caregiver, focusing on the transition to palliative care	*N* = 16 patients Advanced cancer patients no longer receiving potentially curative treatment Patient backgrounds: European Union (*N* = 6), Oceania (*N* = 3), Asia (*N* = 5), Africa (*N* = 2)	Qualitative Data collection: semistructured interviews Data analysis: thematic analysis	There was a lack of understanding of the concept and meaning of palliative care—“what ‘palliative’ meant was, for many participants, lost in translation” Often patients (and caregivers) felt the explanation given by clinicians was not clear Compared to the expectation of home/family-based care, hospital-based palliative care was difficult for many patients to understand. Some patients believed a return home was indicative of the onset of the dying process and that remaining in hospital entailed hope for recovery Patients reported cultural-based differences in treatment expectations and difficulties communicating these expectations Avoiding talking about cancer, death, and dying was a predominant theme and was linked to cultural backgrounds Talking about death and dying or terminal illness was frequently associated by participants with “giving up” or hastening death Faith and religion was a means of coping for many patients, and often religious faith was associated with hope For some patients, religious faith presented a challenge to their doctor’s expertise and authority, with implication that “God knows better”
Ko, 2022	USA, nonprofit cancer organization	To explore ACP experiences, particularly what helps and hampers ACP engagement, and elicit suggestions to improve the ACP process	*N* = 30 patients Hispanic/Latino patients aged > 18 Breast (*N* = 16), prostate (*N* = 3), lung (*N* = 2), thyroid (*N* = 2), and “other” (*N* = 7) cancers	Qualitative Data collection: semistructured interviews Data analysis: thematic analysis	Safeguarding families from having conflicts (i.e., decision-making conflict, emotional distress) was an important reason for ACP engagement Expressing EOL treatment preferences, particularly forgoing or withdrawing life supports, was expected to evoke family’s strong emotional reactions and was a barrier to ACP When the patient is the sole caregiver, protecting family from emotional vulnerability becomes even more important Participants’ experiences with family and friends relating to EOL care helped them accept death and think about values and goals of care, which further propelled them to engage in ACP Cultural taboos on death and dying also led to the family’s lack of participation Patients flights that ACP was not a priority compared to focusing on current medical treatment The ACP process was complicated by family dynamics and family difficulty in accepting death Family members’ avoidance to engage in EOL communication deterred participants’ engagement in ACP Witnessing family suffering intensified patients’ suffering Patients valued ACP education and wanted it offered to family as a form of family support Most patients want physicians to address EOLC with patients and family Physician’s active dialogue in one’s native tongue (e.g., Spanish), beyond providing written documents, was seen as helpful for enhancing patients’ comprehension
Krishnamurthy, 2024	USA, hospital oncology clinics and community-based clinics	To explore qualitative barriers and facilitators to early PC access in an urban setting with Black and Latina women with advanced breast cancer	*N* = 20 patients *N* = 14 Black patients, *N* = 4 Latina patients Stage 3 C or 4 breast cancer patients	Qualitative Data collection: semistructured interviews Data analysis: inductive thematic analysis	Patient-reported barriers to PC access: 1. Fractured communication between patients and providers stemming from patients’ reluctance to initiate conversations/expectation that providers would initiate difficult or uncomfortable conversations 2. Poor health literacy among patients regarding the scope and role of PC 3. Language-related barriers which affected both access and the provider–patient relationship Facilitators to PC access: 1. Providing and receiving community support and advocacy 2. Holistic care, particularly providers/health systems addressing social determinants of health 3. Cultural/ethnic representation of patients among their providers 4. Trust within the provider–patient relationship
Martin, 2011	USA, CanCORS respondents (hospitals, community, health centers)	Comparing willingness to spend financial resources to extend life among Black, Hispanic, Asian, and White participants of the Cancer Care Outcomes Research and Surveillance Consortium (CanCORS) study	*N* = 4214 patients *N* = 3055 White patients, *N* = 626 Black patients, *N* = 317 Hispanic patients, and *N* = 216 Asian patients Colorectal and lung cancer patients	Quantitative Data collection: interviews or self-administered survey Data analysis: descriptive statistics	A higher proportion of Black patients (80%) were willing to spend all personal resources to extend life compared with Whites (54.1%), Hispanics (69.1%), and Asians (72%)
Nedjat-Haiem, 2013	USA, patients receiving public cancer care	To explore the EOL care concerns of 24 Latinas receiving treatment for metastatic cancers	*N* = 24 Latino patients Aged 35–79 years, average age 52 years	Qualitative Data collection: telephone interviews in Spanish Data analysis: thematic analysis	Patients expressed concern about burdening family members with the knowledge of the serious nature of their illness and likelihood of dying Patients were concerned that they would upset their family members by talking about dying and EOL
Nielsen, 2015	Canada, urban health center	To examine the palliative home care experiences of 4 Chinese immigrants with terminal cancer, their family caregivers, home care nurses, and healthcare providers	*N* = 4 patients 2 male, 2 female patients aged 50–80 years *N* = 2 lung, *N* = 1 brain, *N* = 1 liver cancer patients	Qualitative Data collection: interviews and observations Data analysis: ethnographic data analysis	Patients felt there was an [inaccurate] assumption among physicians that death was a taboo topic and dying at home was not preferred Family caregivers, who spoke English, were depended upon as translators
O’Mahony, 2021	USA, outpatient PC trial patients	To evaluate the influence of demographic factors such as religiosity, education, income, race, and ethnicity on treatment preferences for end of life care	*N* = 308 patients Aged > 55 71% White, 22% Black, 6% Hispanic, 7% other minorities	Quantitative Data collection: questionnaire Data analysis: bivariate associations and multiple logistic regression analysis	White patients were more likely to want less invasive or comfort care measures at the end of life and were less likely to want CPR than minority patients
Phipps, 2003	USA, cancer center, hospice program, HCP identified patients	To investigate differences in attitudes, preferences, and behaviors regarding end of life in terminally ill patients and their designated family caregivers	*N* = 68 patients 30 African American patients, 38 White patients Mean age 65 (African American) or 71 (White)	Mixed methods Data collection: interviews and data from medical records Data analysis: Fisher’s exact test, kappa test; coding of interview responses	90% of patients thought that the patient should be the primary decision-maker White patients were more likely to have a living will and a durable power of attorney for healthcare than African American patients The most common reason to have a living will/durable power of attorney for healthcare among minority patients was to exercise control, to avoid confusion about treatment preferences, and to shield loved ones from having to make difficult decisions In the near-death condition, African American patients were more likely to desire all of the life-supportive measures Patients and caregivers often disagreed regarding preferences for or against the three life-sustaining measures
Rosario-Ramos, 2022	USA, oncology clinic	To describe important Latino EOL topics for patient–caregivers coping with cancer	*N* = 7 patients Latinos with a score of > 3 on the Distress Thermometer Aged 40–76 years Stage III/IV cancer *N* = 3 breast, *N* = 2 multiple myeloma, *N* = 1 cervical, *N* = 1 squamous cell cancer patients	Qualitative Data collection: free listing of important topics as defined by patients Data analysis: thematic analysis, deductive content analysis	11/14 of Latino patients expressed the importance of “receiving support from friends and family”
Seto Nielsen, 2013	Canada, urban health center	To describe and examine how meanings of home condition negotiations of care for Chinese immigrants with advanced cancer receiving palliative home care in Toronto, Canada	*N* = 4 patients 2 male, 2 female patients	Qualitative Data collection: open-ended interviews Data analysis: ethnographic data analysis	Palliative care was not viewed as an intrusion that was thrust upon the home without permission or invitation but a necessary insertion into the routines of the home Expressing dissatisfaction was not necessarily a reflection of unhappiness but rather tension of living and dying at home
Seymour, 2007	UK, community or HCP identified patients	To present the experiences and expectations in relation to the provision of end of life care at home and in hospices	Study 1: *N* = 77 patients Study 2: *N* = 92 patients Study 1: *N* = 71 White British, *N* = 2 White Irish, *N* = 2 Black Caribbean, *N *= 1 Black British Study 2: *N* = 39 British Chinese, *N* = 22 Hong Kong Chinese, *N* = 31 mainland Chinese	Qualitative Data collection: focus groups, interviews Data analysis: qualitative coding of interview transcripts	Hospice care was seen as clearly linked to inevitable death but symbolic of the hope for a “good death” among White participants Among Chinese participants, hospice was seen to demonstrate one had become a “burden” to one’s family and that there was no other option for care and was giving up
True, 2005	USA, cancer center, hospice program patients	To explore the role of spiritual coping in EOL preferences	*N* = 68 patients 30 African American patients, 38 White patients Mean age 65 (African American) or 71 (White) Stage IIIb or 4 lung cancer or state IV colon cancer	Mixed methods Data collection: face to face interviews Data analysis: quantitative analysis	When near death, African American patients were more likely to desire all of the life-supportive measures 87% of African American patients as compared to 66% of White patients wanted to be hospitalized if they were near death White patients were more likely to have a living will and a durable power of attorney for healthcare than African American patients

### Study quality

A kappa score of 0.64 was obtained for interrater reliability for assessment of study quality, indicating substantial agreement between reviewers [31]. The quality of included studies varied across assessment domains as seen in Appendix 5. Most studies achieved the highest score possible for criteria appraising underpinning research, statement of research aims, research setting description, appropriate study design, inclusion of recruitment data, and data collection tool appropriateness. The rationale for the data collection tool used and description of the collection process was also generally of high quality, with most studies achieving the highest or second highest score possible. There was a marked lack of stakeholder input into research design, with only three studies providing evidence of stakeholder involvement [38, 42, 51]. Appraisal also identified limited justification for the analysis method, with nearly half of included studies providing no or basic justification. Discussions of study strengths and limitations were of overall high quality, with only three studies providing no or limited discussion.

### Main findings

#### What are the palliative and EOL care needs of patients from CALD backgrounds?

Five studies highlighted staying positive to cope with illness [37] and protect families/caregivers from suffering [32, 36, 38, 41, 47] as a key need reported by African American, Chinese, Hispanic, and Latino/a with advanced cancer. A desire to protect loved ones from difficult choices was a key reason reported by African American and Hispanic patients for engaging with EOL services and creating ACPs. These patients also felt that by making EOL decisions in advance they could also regain control and ensure their choices were adhered to [41, 47]. However, Asian and Hispanic patients were less likely to report receiving needed medications or care compared to White [52] or African American patients [37]*.*

Maintaining autonomy in decision-making during the EOL was another key need reported by Hispanic and African American patients [41, 47]. However, this need for autonomy in EOLC was balanced by a simultaneous need for familial support and involvement with decision-making. Most patients preferred and needed family involvement in their EOLC journey and decision-making. Patients from minority backgrounds were often reported to be dissatisfied with their treating team when there was a failure to proactively involve family members in key conversations [32]. Patients also reported a need for healthcare providers to look past assumptions about patients’ cultures or implicit biases [40] to focus more on individual patient attitudes towards death and dying [49]. Trust in the physician–patient relationship was another key need [32, 36, 42], and one often emphasized to Black and Latina patients by the lack of representation of their cultural background among their providers [42]. Patients also wanted to have a safe space to discuss EOLC options and were grateful when healthcare providers created these opportunities [36].

#### What are the palliative and EOL care preferences of patients from CALD backgrounds?

Preference for comfort care among participants from CALD backgrounds in the included studies varied. One study exploring views of African American, Hispanic, and Asian patients identified that they were more likely to prefer life-extending treatments, even if needing to expend all financial resources to do so. This preference was less common if patients were older, had multiple dependents, or believed they had less than 5 years to live [42]. As noted by O’Mahony et al., patients with a greater awareness of the terminal nature of their illness were more likely to prefer supportive care [46].

Preferences of people from CALD backgrounds for the continued role of healthcare providers in making decisions about PC or EOLC varied; some preferred physicians to be the primary decision-maker [34] and reported a lack of clinician initiative as a barrier to EOLC access [41, 42], while others preferred that they themselves take the lead [47]. Regardless of the level of clinician initiative preferred, overall, patients preferred to make the decision to disclose information about diagnosis and prognosis to their family/caregiver themselves [32]. Compared to African American patients, White patients were more likely to have discussed EOLC preferences and have living wills or do-not-resuscitate orders [35, 47]. Despite a greater perceived need for hospice among African American patients, they were less likely to choose hospice services [33]. Access to support groups as well as the opportunity to provide support and advocate for other cancer patients’ access to PC services was valued among some Black and Latina patients [42].

#### Factors which influenced care preferences

**Fig. 2:**
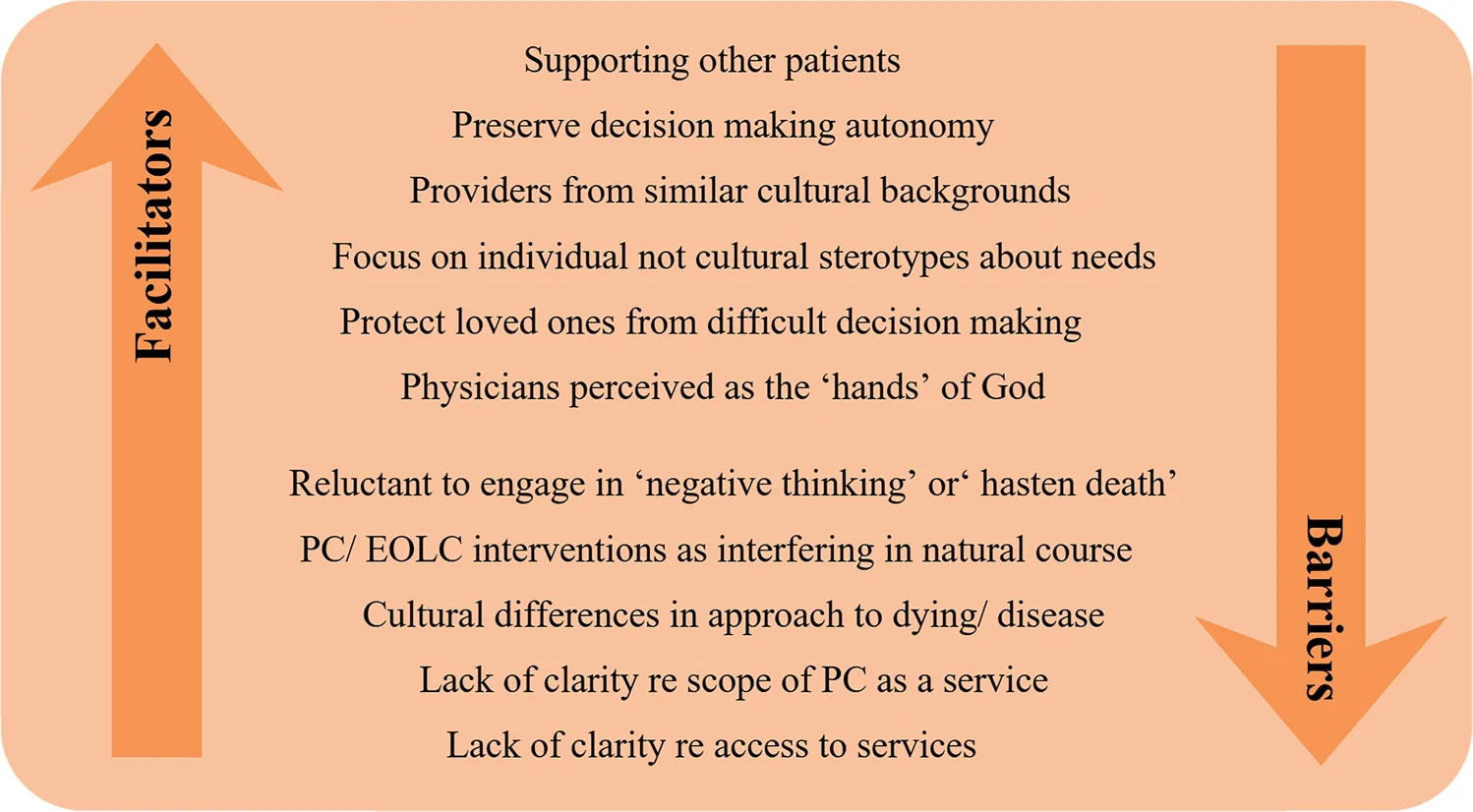
Factors which influence service engagement

A variety of factors were identified which either helped or hindered patients when engaging with EOL and PC services. Common themes were faith, family, and systems familiarity. The interplay between these factors can be seen in Fig. 2.

#### Family

Family involvement was seen as an essential part of engaging with EOL and PC services among patients from CALD backgrounds [32, 39, 47, 48]. Some African and Hispanic patients sought EOLC to preemptively support their families through the dying process [41, 47]. Conversely, other studies found that families’ reluctance to engage in “negative thinking” was a barrier to patients utilizing PC services [40, 41]. As noted by multiple authors [36, 44], Hispanic patients were often reluctant to engage in EOLC conversations due to family resistance and often downplayed their struggles and desire for support so as to not “burden” their family. As reported by Phipps [47], among African Americans, discrepancies between patient and family/caregiver preferences for EOLC and life-sustaining treatment were also found, and these disagreements were usually not able to be resolved with discussion.

#### Faith and spirituality

Faith and spirituality had a varied influence on patient preferences and needs for EOL or PC. This systematic review found that African American patients were more likely to draw on their spirituality to cope with their cancer prognosis. Among African American, Asian, Pacific Islander, and Hispanic patients, religious/spiritual coping and faith were often reported as a barrier when seeking PC. This was rooted in beliefs of “divine intervention” [40, 51], “God provid[ing] a cure” [32], or non-life-sustaining treatment would be acting against “God’s will” [39]. Among these patients, there was a strong preference for aggressive care [33, 46, 51]. However, as noted by Campbell et al. [32] and Glaser et al. [36], among African American and Latina patients who believed their physicians to be the “hand of God,” the preference was to follow their suggestion to utilize PC.

#### Clarity about how and when to access services

Many CALD patients expressed confusion about requirements for accessing palliative care services. Multiple studies reported that patients and their families felt patients were not “sick enough” [32, 47] or “poor enough” [33, 34] to access services or subsidies. Exacerbating this misunderstanding was an unfamiliarity with the range and flexibility of services [34, 42]. A lack of interpreter services as well as a lack of a direct/inadequate translation for palliative care terminology and ethos [36, 40] left patients feeling “isolated and frustrated” [40] or that they “were not being heard” [42]. It also highlighted misunderstandings regarding the role of palliative and EOLC. Among Maori, Pacific Islander, and Asian patients, there was a belief that hospice was only a place, rather than both an institution as well as a philosophy of treatment [34], a belief that does not align with service provision in New Zealand. Among patients from various CALD backgrounds, the role of PC throughout the care journey was unclear and often misunderstood as equivalent to EOLC [43]or mutually exclusive from active treatment. Engagement with PC services was at times felt by CALD patients to imply “giving up” [38, 40, 50], “hastening” death [37, 43], or “foreclosing on treatment possibilities” [39].

## Discussion

This systematic review provided evidence on the EOL and PC needs and preferences of cancer patients from CALD backgrounds. For many, a lack of culturally/linguistically appropriate information about the role and breadth of services, as well as how to access them, was a fundamental barrier. Among others, cultural beliefs about family and death hindered service engagement. Interestingly, for some patients these beliefs encouraged service utilization. Compared to White patients, patients from African American backgrounds were more likely to prefer life-sustaining care and less likely to have discussed EOL options. Despite willingness to expend financial resources to sustain life, Asian and Hispanic patients were less likely to report receiving needed or satisfactory care.

This systematic review found family/caregiver involvement to be both a facilitator and a barrier to service engagement. Compared to patients from a non-CALD background, patients from CALD backgrounds often desire a higher degree of family involvement [53]. EOL service utilization simultaneously allowed patients to protect their families from difficult decision-making while exerting a degree of control over their future, especially when discrepancies arose between patient and family preferences for care. For others however, family involvement was a barrier. A desire to avoid “negative thinking,” a cultural reluctance to discuss death/dying [54], or an unwillingness to dampen their own or their family’s positive outlook with the “reality” of their prognosis often hindered engagement with services. This reluctance was often compounded if patients held religious/spiritual beliefs.

The varied role of religion at the EOL is well reported [55] and aligns with the results of this review. Some patients believed physicians acted as the “hand of God” and thus were encouraged to pursue EOLC. Beliefs against utilizing EOLC may have been exacerbated by misunderstandings about the role of EOL and PC. This review highlighted a confusion among many CALD patients about the meaning and place of PC within cancer management, with PC often seen as an alternative to active cancer treatment rather than an essential adjunct. Additionally, many patients and families believed there to be a disease or wealth threshold to be met to access PC/EOLC. This lack of accurate knowledge about the depths and variations of PC is not limited to this study, with a global deficit identified in the literature [56]. CALD patients’ incomplete or inaccurate understanding may however be reflective of a larger issue.

Misunderstandings about the how and why of PC use may be compounded by a lack of adequate and accurate translated information available to patients and their families. This review identified a paucity of both easy and timely access to interpreters. Furthermore, even when utilized, there were difficulties providing equivalent culturally congruent translations of PC terms. CALD patients often lack adequate access to cancer resources, often compounded by a lack of English proficiency and availability of resources in a patient’s first language [57]. However, it is important to consider that challenges in translating palliative and EOLC concepts across languages may in part stem from their grounding in Western cultural value [58], with the absence of equivalent terms reflective of differences in how care, decision-making, and the end of life are conceptualized cross-culturally.

### Implications

The EOL is a challenging time for cancer patients, and provision of tailored and timely care is essential. PC is an essential component of EOLC that seeks to relieve physical, psychosocial, and/or spiritual suffering of patients and families [2]; however, integration of PC into patient management is lacking [58]. A shift in both institutional and individual attitudes is needed to facilitate PC as a standard component of patient care, rather than only as part of EOLC. As demonstrated by Hoerger et al., in their randomized controlled trial of 598 cancer patients, psychoeducation increased patient preferences for early PC [59]. Development and integration of institutional policies which clearly outline educational resources for both staff and consumers on both the role of PC and pathways to access could address the barriers highlighted in this review. Furthermore, development of educational materials should involve consumers so that they are more relevant to their target populations.

Access to accurate and understandable PC information is an essential part of health equity and informed decision-making [60]. The paucity of these resources is a key contributor to disparities in both patient uptake and satisfaction with healthcare [56]. System changes to design and distribute these resources are needed to address the gap. Consultation and collaboration with community groups/leaders during this process may be beneficial to better provide materials which align with the needs and preferences of those they aim to support.

Healthcare providers need to be equipped to recognize and respond to patient needs to provide quality care. Although providers often define patient needs from a clinical perspective, elucidating patients’ own reports offers valuable insights into what matters to them and the priorities that shape their choices. This review identified a pronounced lack of studies investigating patient needs. Among studies that did identify patient-reported needs, this was often the result of open interview questions as opposed to directly asking patients what they believe they need. There is a need for future research to focus specifically on patient-identified EOL and PC needs. Doing so may also aid researchers, and consequently care providers, in better exploring patients’ current understanding of services and elucidating care priorities from a culturally congruent context. Additionally, there was a dearth of studies exploring experiences with VAD conversations and pathways. Given the growing legalization of VAD [61], research into patient expectations and experiences seeking VAD is needed to tailor services to align with patient desires.

However, as demonstrated by the results of this study, preferences between and importantly within CALD groups are not monolithic, and what facilitates engagement for one individual may constitute a barrier for another. Insights from CALD groups can guide culturally sensitive care; however, they should not be extrapolated to all individuals within a cultural or ethnic group, and assumptions based on cultural background alone should be avoided. As such, providers should use these themes as a starting point for awareness and conversations with patients, rather than prescriptive conclusions.

### Strength and limitations

This systematic review has a number of key strengths. This study was developed and conducted in line with PRISMA guidelines and registered prospectively with PROSPERO. A comprehensive search strategy across five databases was developed with the aid of a university research librarian. Multiple authors were involved in data screening and quality appraisal, with disagreements discussed between authors until consensus was achieved.

However, despite its strengths, there were also limitations. This systematic review excluded gray literature which may have led to omission of practice points, policy, or community feedback, which, though not peer-reviewed, may have enriched understanding of the topic or future research directions. This study also excluded non-English studies and consequently may have excluded relevant literature published in other languages. Indigenous Australians were excluded from this study. Notably, they are also often excluded from the definition of CALD people in broader literature and policy. Beyond a limitation of this study, it also raises a potential need to redefine these terms and approaches to research. Of the twenty studies included, only five were conducted outside the USA. This is notable given the differences in healthcare costs and accessibility between the USA, where there is only limited access to state-funded healthcare and high out-of-pocket costs for patients [55], and other countries such as Australia or the UK, where there is universal government-funded healthcare for citizens. Additionally, most studies focused on African American or Hispanic populations, with only a minority of studies exploring the interactions of patients from other backgrounds, which is of note given the difference in the ethnicity profile of the USA compared to other countries like Australia [62, 63].

## Conclusion

This review illustrates the complex and varied needs and preferences of patients from CALD backgrounds in relation to EOL and PC. Common themes emerged across studies; however, their influence differed between individuals and groups, serving as both facilitators and barriers to positive service interactions. These findings underscore the importance of avoiding cultural generalizations and the need for individualized, culturally responsive care. System-level changes, including increased messaging on the role and flexibility of palliative care, and improved access to culturally/linguistically appropriate resources are needed. Ultimately, meaningful engagement with CALD communities and patients remains critical to ensuring care aligns with patients’ values and expectations.

## Supplementary Information

Below is the link to the electronic supplementary material.

ESM 1 (PFD 131 KB)

ESM 2 (DOCX 23.2 KB)

ESM 3 (DOCX 22.7 KB)

ESM 4 (PDF 90.8 KB)

ESM 5 (PDF 66.3 KB)

## Data Availability

No datasets were generated or analysed during the current study.
