# Supplementary material for: What are the palliative and end-of-life care needs and preferences of people with cancer from culturally and linguistically diverse (CALD) backgrounds?: A systematic review
Source: Support Care Cancer. 2026 Oct 29;34(11):1171. doi: 10.1007/s00520-026-11341-2 (PMC13719822; doi:10.1007/s00520-026-11341-2)
Supplement: Supplementary file 2 [file 520_2026_11341_MOESM4_ESM.pdf]

Appendix 3. QuADS Criteria

QuADS Criteria	0	1	2	3
1. **Theoretical or conceptual underpinning to the research**	No mention at all.	General reference to broad theories or concepts that frame the study. e.g. key concepts were identified in the introduction section.	Identification of specific theories or concepts that frame the study and how these informed the work undertaken. e.g. key concepts were identified in the introduction section and applied to the study.	Explicit discussion of the theories or concepts that inform the study, with application of the theory or concept evident through the design, materials and outcomes explored. e.g. key concepts were identified in the introduction section and the application apparent in each element of the study design.
**2. Statement of research aim/s**	No mention at all.	Reference to what the sought to achieve embedded within the report but no explicit aims statement.	Aims statement made but may only appear in the abstract or be lacking detail.	Explicit and detailed statement of aim/s in the main body of report.
**3. Clear description of research setting and target population**	No mention at all.	General description of research area but not of the specific research environment e.g. 'in primary care.'	Description of research setting is made but is lacking detail e.g. 'in primary care practices in region [x]'.	Specific description of the research setting and target population of study e.g. 'nurses and doctors from GP practices in [x] part of [x] city in [x] country.'
**4. The study design is appropriate to address the stated research aim/s**	No research aim/s stated or the design is entirely unsuitable e.g. a Y/N item survey for a study seeking to undertake exploratory work of lived experiences. .	The study design can only address some aspects of the stated research aim/s e.g. use of focus groups to capture data regarding the frequency and experience of a disease.	The study design can address the stated research aim/s but there is a more suitable alternative that could have been used or used in addition e.g. addition of a qualitative or	The study design selected appears to be the most suitable approach to attempt to answer the stated research aim/s.

			quantitative component could strengthen the design.	
**5. Appropriate sampling to address the research aim/s**	No mention of the sampling approach.	Evidence of consideration of the sample required e.g. the sample characteristics are described and appear appropriate to address the research aim/s.	Evidence of consideration of sample required to address the aim. e.g. the sample characteristics are described with reference to the aim/s.	Detailed evidence of consideration of the sample required to address the research aim/s. e.g. sample size calculation or discussion of an iterative sampling process with reference to the research aims or the case selected for study.
**6. Rationale for choice of data collection tool/s**	No mention of rationale for data collection tool used.	Very limited explanation for choice of data collection tool/s. e.g. based on availability of tool.	Basic explanation of rationale for choice of data collection tool/s. e.g. based on use in a prior similar study.	Detailed explanation of rationale for choice of data collection tool/s. e.g. relevance to the study aim/s, co-designed with the target population or assessments of tool quality.
**7. The format and content of data collection tool is appropriate to address the stated research aim/s**	No research aim/s stated and/or data collection tool not detailed.	Structure and/or content of tool/s suitable to address some aspects of the research aim/s or to address the aim/s superficially e.g. single item response that is very general or an open-response item to capture content which requires probing.	Structure and/or content of tool/s allow for data to be gathered broadly addressing the stated aim/s but could benefit from refinement. e.g. the framing of survey or interview questions are too broad or focused to one element of the research aim/s.	Structure and content of tool/s allow for detailed data to be gathered around all relevant issues required to address the stated research aim/s.
**8. Description of data collection procedure**	No mention of the data collection procedure.	Basic and brief outline of data collection procedure e.g. 'using a questionnaire distributed to staff'.	States each stage of data collection procedure but with limited detail or states some stages in detail but	Detailed description of each stage of the data collection procedure, including when, where and how

			omits others e.g. the recruitment process is mentioned but lacks important details.	data was gathered such that the procedure could be replicated.
**9. Recruitment data provided**	No mention of recruitment data.	Minimal and basic recruitment data e.g. number of people invited who agreed to take part.	Some recruitment data but not a complete account e.g. number of people who were invited and agreed.	Complete data allowing for full picture of recruitment outcomes e.g. number of people approached, recruited, and who completed with attrition data explained where relevant.
**10. Justification for analytic method selected**	No mention of the rationale for the analytic method chosen.	Very limited justification for choice of analytic method selected. e.g. previous use by the research team.	Basic justification for choice of analytic method selected e.g. method used in prior similar research.	Detailed justification for choice of analytic method selected e.g. relevance to the study aim/s or comment around of the strengths of the method selected.
**11. The method of analysis was appropriate to answer the research aim/s**	No mention at all.	Method of analysis can only address the research aim/s basically or broadly.	Method of analysis can address the research aim/s but there is a more suitable alternative that could have been used or used in addition to offer a stronger analysis.	Method of analysis selected is the most suitable approach to attempt answer the research aim/s in detail e.g. for qualitative interpretative phenomenological analysis might be considered preferable for experiences vs. content analysis to elicit frequency of occurrence of events.
**12. Evidence that the research stakeholders have been considered in research design or conduct.**	No mention at all.	Consideration of some the research stakeholders e.g. use of pilot study with target sample but no	Evidence of stakeholder input informing the research. e.g. use of pilot study with feedback influencing the study	Substantial consultation with stakeholders identifiable in planning of study design and in preliminary work e.g. consultation in the

		stakeholder involvement in planning stages of study design.	design/conduct or reference to a project reference group established to guide the research.	conceptualisation of the research, a project advisory group or evidence of stakeholder input informing the work.
**13. Strengths and limitations critically discussed**	No mention at all.	Very limited mention of strengths and limitations with omissions of many key issues. e.g. one or two strengths/limitations mentioned with limited detail.	Discussion of some of the key strengths and weaknesses of the study but not complete. e.g. several strengths/limitations explored but with notable omissions or lack of depth of explanation.	Thorough discussion of strengths and limitations of all aspects of study including design, methods, data collection tools, sample & analytic approach.
