# Supplementary material for: What are the palliative and end-of-life care needs and preferences of people with cancer from culturally and linguistically diverse (CALD) backgrounds?: A systematic review
Source: Support Care Cancer. 2026 Oct 29;34(11):1171. doi: 10.1007/s00520-026-11341-2 (PMC13719822; doi:10.1007/s00520-026-11341-2)
Supplement: Supplementary file 3 [file 520_2026_11341_MOESM5_ESM.pdf]

Appendix 5 - Quality Assessment Results

	Factors that impact end-of-life decision making in African Americans with advanced cancer.	Race, treatment preferences, and hospice enrollment: eligibility criteria may exclude patients with the greatest needs for care.	Where do I go from here'? A cultural perspective on challenges to the use of hospice services.	End-of-life treatment preferences: a key to reducing ethnic/racial disparities in advance care planning?	Experiences of Advanced Breast Cancer Among Latina Immigrants: A Qualitative Pilot Study.	The health care experience of patients with cancer during the last year of life: Analysis of the SEER-CAHPS data set	Barriers and Facilitators to Advance Care Planning among Chinese Patients with Advanced Cancer and Their Caregivers.	Racial and ethnic differences in beliefs about lung cancer care.	It doesn't exist...: negotiating palliative care from a culturally and linguistically diverse patient and caregiver perspective.	Rural Hispanic/Latino cancer patients' perspectives on facilitators, barriers, and suggestions for advance care planning: A qualitative study.	"If we don't speak the language, we aren't offered the same opportunities": Qualitative perspectives of palliative care access for women of color living with advanced breast cancer	Racial variation in willingness to trade financial resources for life-prolonging cancer treatment.	Psychosocial Concerns among Latinas with Life-Limiting Advanced Cancers.	Patient-centered care or cultural competence: Negotiating palliative care at home for Chinese Canadian immigrants.	Association of Race with End-of-Life Treatment Preferences in Older Adults with Cancer Receiving Outpatient Palliative Care.	Approaching the end of life: attitudes, preferences, and behaviors of African-American and white patients and their family caregivers.	Important End-of-Life Topics among Latino Patients and Caregivers Coping with Advanced Cancer.	Maintaining distance from a necessary intrusion: A postcolonial perspective on dying at home for Chinese immigrants in Toronto, Canada.	Focus group findings about the influence of culture on communication preferences in end-of-life care.	Treatment preferences and advance care planning at end of life: the role of ethnicity and spiritual coping in cancer patients.
	Campbell CL; Wi	Fishman J; O'Dw	Frey, Rosemary;	Garrido, Melissa	Glaser, Johanna;	Halpern M.T.; Ur	Jia, Zhimeng; Ye	Jonnalagadda S;	Kirby, Emma; Lw	Ko, Eunjeong; Ke	Krishnamurthy N	Martin MY; Pisu I	Nedjat-Haiem, Fr	Nielsen, Lisa Set	O'Mahony, Sean;	Phipps E; True G	Rosario-Ramos,	Seto Nielsen, Lis	Shrank, William	True, Gala
**Theoretical or conceptual underpinning to the research**	3	3	3	3	3	3	3	3	3	2	3	3	3	3	3	3	3	3	3	3
**Statement of research aims**	3	3	3	3	3	3	3	3	3	3	3	3	3	3	3	3	3	3	3	3
**Clear description of research setting and target population**	3	2	3	3	3	2	3	3	3	3	3	3	2	3	3	2	2	3	3	3
**The study design is appropriate to address the stated research aims**	3	3	3	3	3	3	3	3	3	3	3	3	3	2	3	2	3	2	2	3
Appropriate sampling to address the research aims	3	2	2	2	2	3	2	3	3	2	2	2	2	2	2	2	2	2	2	2
**Rationale for choice of data collection tools**	2	3	3	2	2	3	2	3	3	1	3	2	0	3	2	3	3	3	0	3
**the format and content of data collection tool is appropriate to address the stated research aims**	3	2	3	3	3	3	3	3	3	3	3	2	2	3	2	2	2	3	2	3
**Description of data collection procedure**	3	3	3	3	3	3	3	2	3	2	3	2	2	2	1	2	2	2	2	3
**Recruitment data provided**	3	3	3	2	2	3	3	3	3	2	3	2	2	2	2	3	2	2	3	3
**justification for analytic method provided**	2	3	2	2	0	0	2	0	2	0	3	2	0	3	2	1	0	3	0	2
The method of analysis was appropriate to answer the research aims	2	2	2	2	2	2	3	2	3	2	2	2	2	2	3	2	2	2	2	2
**Evidence that the research stakeholders have been considered in research design or conduct**	0	0	0	0	0	0	1	0	0	0	1	0	0	0	0	2	0	0	0	1
Strengths and liitations critically discussed	3	3	2	2	2	2	3	3	2	2	2	2	0	2	2	1	0	1	2	2
	33	32	32	30	28	30	34	31	34	25	34	28	21	30	28	28	24	29	24	33
