# Supplementary material for: What are the palliative and end-of-life care needs and preferences of people with cancer from culturally and linguistically diverse (CALD) backgrounds?: A systematic review
Source: Support Care Cancer. 2026 Oct 29;34(11):1171. doi: 10.1007/s00520-026-11341-2 (PMC13719822; doi:10.1007/s00520-026-11341-2)
Supplement: Supplementary file 4 [file 520_2026_11341_MOESM2_ESM.docx]

**APPENDIX 2 - FULL SEARCH STRATEGY**

**Search terms for culturally and linguistically diverse populations**

cultural diversity/ or "diversity, equity and inclusion"/

Refugee/

Ethnic group/

Minority group/

"cultur* and linguistic* divers*"

(CALD or non-english speak* or ethnic minorit* or ethnic group* or ethnic population* or ethnic

diversit* or migrant* or immigrant) or "hispanic american*" or "mexican american*

**Search terms for palliative/ end -of -life care**

cancer palliative therapy/ or palliative therapy/ or terminal care/ or hospice care/

terminally ill patient/ or Attitude to Death/

"end of life" or terminally ill or hospice* or palliative* or EOL or "dying at home" or beareavement

care or hospice care*

(terminal adj1 (ill* or care or carer* or caring or wishes or preference*)

bereavement/ or grief

**Search terms for cancer**

exp neoplasm/

(neoplasm* or cancer* or tumor* or tumour*)

**Search terms for preferences, needs and experiences**

caring or wishes or preference*

**Search terms for voluntary assisted dying**

euthanasia OR "assisted suicide" OR "right to die" OR "assisted dying" OR "voluntary assisted

dying" OR vad OR "physician assisted dying" OR "medical assisted dying" OR "aid in dying" )
