# Supplementary material for: What are the palliative and end-of-life care needs and preferences of people with cancer from culturally and linguistically diverse (CALD) backgrounds?: A systematic review
Source: Support Care Cancer. 2026 Oct 29;34(11):1171. doi: 10.1007/s00520-026-11341-2 (PMC13719822; doi:10.1007/s00520-026-11341-2)
Supplement: Supplementary file 5 [file 520_2026_11341_MOESM3_ESM.docx]

**APPENDIX 2 - INCLUSION & EXCLUSION CRITERIA**

**Inclusion criteria**

- Racial/ ethnic minority population groups (e.g., African Americans, Hispanic, Asian, etc)
- End of life/ palliative care services including hospice, advance care directives, VAD
- Population centred in OECD countries
- Patient needs/ preferences/ experiences/ opinions
- Patients with cancer diagnosis seeking or utilising end of life/ palliative care services

**Exclusion criteria**

- Non OECD countries (results non transferable to Australian context)
- Paediatric, <18yo
- Non English language
- Commentaries/ editorials (i.e., non peer reviewed articles)
- Grey literature
- Non primary studies e.g., literature reviews
- Case studies
